# Reappraisal of fibrosis-4 index and non-alcoholic fatty liver disease fibrosis score for advanced fibrosis in average-risk population

**DOI:** 10.3389/fmed.2022.1024836

**Published:** 2022-11-03

**Authors:** Huiyul Park, Eileen L. Yoon, Mimi Kim, Jonghyun Lee, Seon Cho, Dae Won Jun, Eun-Hee Nah

**Affiliations:** ^1^Department of Family Medicine, Myongji Hospital, Hanyang University College of Medicine, Seoul, South Korea; ^2^Department of Internal Medicine, Hanyang University College of Medicine, Seoul, South Korea; ^3^Hanyang Institute of Bioscience and Biotechnology, Hanyang University, Seoul, South Korea; ^4^Department of Radiology, Hanyang University College of Medicine, Seoul, South Korea; ^5^Department of Translational Medicine, Hanyang University Graduate School of Biomedical Science and Engineering, Seoul, South Korea; ^6^Department of Laboratory Medicine, Health Promotion Research Institute, Seoul, South Korea

**Keywords:** advanced hepatic fibrosis, fibrosis-4 index, non-alcoholic fatty liver disease fibrosis score, magnetic resonance elastography, average-risk population

## Abstract

**Background and aim:**

The current cut-offs for fibrosis-4 (FIB-4) and non-alcoholic fatty liver disease fibrosis score (NFS) are suboptimal for screening because of low accuracy and high false-negative rates in average-risk populations. This study aimed to reappraisal the performance of FIB-4 and NFS in such average-risk populations.

**Methods:**

This is a cross-sectional study, which retrospectively reviewed the magnetic resonance elastography (MRE) data of 8,522 subjects. Individuals with history of significant alcohol consumption and those with positive viral serologic markers were excluded. Finally, 6,215 average-risk individuals were analyzed.

**Results:**

The area under the receiver operating characteristic curves (AUROCs) of FIB-4 for the diagnosis of advanced hepatic fibrosis was higher than that in the NFS especially in the metabolically healthy. The AUROCs of FIB-4 for in the average-risk population was also higher than that in the NFS (0.840 in FIB-4 vs. 0.798, *P* = 0.036). However, the sensitivity of FIB-4 and NFS was low (69.6 and 61.4%, respectively) in applying the current cut-off of FIB-4 [1.3 (2.0)] and NFS [-1.455 (0.12)]. At cut-off of FIB-4 at 1.0, sensitivity (90.2%), and negative predictive value (99.7%) were improved.

**Conclusion:**

The diagnostic performance of FIB-4 was better than that of NFS for screening hepatic fibrosis in average-risk populations. It is recommended to use FIB-4 rather than NFS, when screening for hepatic fibrosis in general population.

## Introduction

Socioeconomic burden of non-alcoholic fatty liver disease (NAFLD) has been increasing of late (1, 2). The most important risk factor for liver-related and overall mortality is the stage of hepatic fibrosis in patients with NAFLD (3, 4). Therefore, it is very important to screen and evaluate for the presence and severity of hepatic fibrosis in such patients (5–7). Prevalence of significant and advanced hepatic fibrosis in the general population is 5.1–9.5 and 1.3–2.6%, respectively (8, 9). Therefore, early detection of hepatic fibrosis in the general population is necessary to prevent liver-related events and to decrease medical costs. However, as no optimal screening algorithms and effective treatment for patients with NAFLD have yet been developed, screening for significant or advanced hepatic fibrosis is not recommended for general population.

Notably, health check-ups are extensively conducted for average-risk populations. Sonographic fatty liver and elevated liver enzyme levels are the most common reasons for referring patients from primary care centers to referral centers. However, the sensitivity of ultrasonography or biochemical tests for significant or advanced hepatic fibrosis is unsatisfactory. In recent times, the fibrosis-4 (FIB-4) index and non-alcoholic fatty liver disease fibrosis score (NFS) tests have become common as the first step to screen advanced hepatic fibrosis in individuals with known viral hepatitis and NAFLD (10). However, only one relevant large-scale study has been conducted for screening hepatic fibrosis in the general population (11). The study reported that FIB-4 and NFS were suboptimal for screening purposes because of the high risk of over-diagnosis in the general population (11). Therefore, special caution is needed when using the current low cut-off of FIB-4 or NFS as a single screening strategy in average-risk populations. New non-invasive methods or optimization of FIB-4 or NFS are needed for detecting fibrosis in low-prevalence fibrosis settings. However, the variables used in FIB-4 and NFS are generally already included in health check-up tests, and therefore, they do not require additional tests in primary care setting. Recent guidelines recommend to use FIB-4 and NFS over other non-invasive fibrosis markers as a first step screening tools, because they are mort well validated and have shown the best diagnostic accuracy among non-invasive fibrosis markers (10, 12). Moreover, considering that other tests (e.g., transient elastography) after FIB-4 and NFS are sequentially applied for the advanced hepatic fibrosis screening algorithm, FIB-4 and NFS tests as a first step are still attractive screening methods in primary care settings.

New cut-off or optimization of FIB-4 and NFS to reduce false-negatives and increase accuracy is needed for detecting fibrosis in low-prevalence fibrosis settings. This study is an attempt to conduct reappraisal the performance of FIB-4 or NFS and discuss the reasonable cut-off in average-risk populations.

## Materials and methods

### Study design

This was a retrospective, cross-sectional study. The subjects underwent magnetic resonance elastography (MRE) as part of their health check-up. The health check-up program was uniformly performed at 13 nationwide health-promotion centers. This study was approved by the institutional review board (IRB No. HY-2021-04-001-001) of Hanyang University Hospital, and the requirement for informed consent was waived.

### Rationale for abdominal sonography and magnetic resonance elastography as health check-up

Abdominal sonography is one of the basic and most commonly performed health check-up examinations. It can be conducted either when the examinees prefer so or as part of an obligatory basic exam conducted every 1 or 2 years by companies under the Act of Employment in Korea. However, the MRE test is not included in the routine health check-up program. It is offered to patients as an additional option under their own expense. Patients with known chronic liver diseases are managed under a separate health check-up program run by the National Health Insurance Service in Korea. Therefore, they are less likely to be included in this study.

### Inclusion and exclusion criteria

**FIGURE 1:**
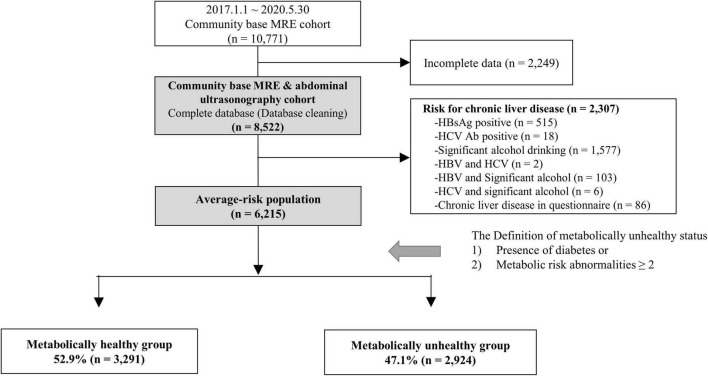
Study flow. MRE, magnetic resonance elastography.

A total of 10,771 subjects who underwent MRE as part of their health check-up examination between January 1, 2017 and May 30, 2020 were included. Among them, 2,249 subjects were excluded owing to missing data on abdominal sonography and clinical information (e.g., metabolic risk abnormalities and variables used in FIB-4 and NFS). Individuals with history of significant alcohol consumption (≥210 g per week for men and ≥ 140 g per week for women) or chronic liver disease in the questionnaire and those with positive result in viral serologic markers (HBsAg and HCV Ab) were also excluded (*n* = 2,307) (Figure 1).

### Clinical parameters of the subjects

Routine health check-up questionnaires were used to obtain the history of hypertension, diabetes mellitus (DM), or dyslipidemia and intake of the corresponding medications for these conditions, as well as the social history of alcohol intake. Anthropometric measurements included waist circumference, blood pressure, height, weight, total fat mass, and lean mass. Additionally, fasting serum glucose, total cholesterol, low-density lipoprotein cholesterol, high-density lipoprotein cholesterol, triglycerides, AST, ALT, and γ-glutamyl transferase levels were measured.

FIB-4 and NFS were calculated, and their low cutoff values of FIB-4 [1.3 (2.0)] and NFS [–1.455 (0.12)] were selected according to the method described by McPherson et al. (13). If age of subjects was 65 or more, the cutoff values of FIB-4 (2.0) and NFS (0.12) were used. If age of subjects was lower than 65, the cutoff values of FIB-4 (1.3) and NFS (–1.455) were used.

### Definition of metabolically healthy and unhealthy groups

We defined the metabolic risk abnormalities as follows (14): (1) waist circumference ≥ 85 cm for women and ≥ 90 cm for men, (2) blood pressure ≥ 130/85 mmHg and/or taking hypertension medication, (3) serum triglyceride ≥ 150 mg/dL, (4) high-density lipoprotein cholesterol < 50 mg/dL for women and < 40 mg/dL for men, and (5) fasting glucose level ≥ 100 mg/dL with HbA1c ≥ 5.7% and/or taking diabetes medication. The metabolically unhealthy group was defined as having diabetes or two or more metabolic risk abnormalities as per our previous study (15). The metabolically healthy group was defined as those with no diabetes and only one or no metabolic risk abnormality.

### Assessment of fatty liver and hepatic fibrosis severity

The presence of fatty liver was evaluated using ultrasonography. Severity was graded as normal, mild, moderate, or severe according to the degree of fat infiltration (16). Liver echotexture, attenuation, and visualization of the intrahepatic vessel borders and/or the diaphragm were used as the indices. Liver stiffness was measured by MRE. All MRE examinations were performed on MRE hardware (GE Healthcare, Waukesha, Wisconsin, USA) with a 1.5-T imaging system using a two-dimensional MRE protocol (17). The acquired MRE images were automatically processed. Liver stiffness was assessed by a radiologist using regions of interest, excluding the vessels. The cut-off values for significant and advanced hepatic fibrosis were set at MRE values of ≥ 3.0 kPa (F2) and ≥ 3.6 kPa (F3), respectively (18). We used various cut-off values for advanced hepatic fibrosis for sensitivity analysis. The range of advanced fibrosis was defined as MRE values of 3.2–4.0 kPa for sensitivity analysis (19–23).

### Statistical analyses

Continuous and categorical variables are presented as mean ± standard deviation and as numbers and percentages, respectively. Categorical variables were analyzed using either the χ^2^-test or Fisher’s exact test, whereas continuous variables were analyzed using Student’s independent *t*-test. Statistical analyses were performed using SPSS version 26.0 for Windows (SPSS Inc., Chicago, IL, USA). The area under the receiver operating characteristic (AUROC) curves of FIB-4 and NFS were compared using DeLong’s test in MedCalc version 20 (MedCalc Software Ltd., Ostend, Belgium). Statistical significance was set at *P* < 0.05.

## Results

### Baseline characteristics

A total of 6,215 average-risk subjects, who did not have chronic viral hepatitis and significant alcohol intake, were identified as the average-risk group for this study and were analyzed (Figure 1). These subjects had 26.4, 9.2, and 23.2% prevalence of hypertension, diabetes, and metabolic syndrome, respectively (Table 1). The prevalence of sonographic fatty liver in total, metabolic healthy, and unhealthy group was 47.7, 31.1, and 66.3%, respectively. The prevalence of significant and advanced hepatic fibrosis diagnosed according to MRE findings in this average-risk group was 6.6 and 1.6%, respectively. The proportion of metabolically healthy individuals in this average-risk group was 52.9%. The prevalence of significant and advanced hepatic fibrosis in the metabolically healthy group was 3.9 and 0.8%, respectively.

**TABLE 1:** Baseline characteristics of the average-risk population according to metabolic status.

	Total subjects (*n* = 6,215)	Metabolically healthy group (*n* = 3,291)	Metabolically unhealthy group (*n* = 2,924)	*P*-value
Age (years)^†^	47.5 ± 10.1	45.9 ± 10	49.3 ± 10	<0.001
Male	5,031 (80.9)	2,514 (76.4)	2,517 (86.1)	<0.001
Hypertension	1,642 (26.4)	279 (8.5)	1,363 (46.6)	<0.001
Diabetes	571 (9.2)	0 (0)	571 (19.5)	<0.001
Alcohol consumption (g/week)^†^	57.2 ± 0.7	38.1 ± 55.3	42.8 ± 59.3	0.001
Number of metabolic risks^†^	1.52 ± 1.29	0.49 ± 0.5	2.68 ± 0.87	<0.001
Metabolic syndrome	1,442 (23.2)	0 (0)	1,442 (23.2)	<0.001
BMI (kg/m^2^)^†^	24.8 ± 3.2	23.4 ± 2.5	26.3 ± 3.1	<0.001
Waist circumference (cm)^†^	85.5 ± 9	81.2 ± 7.6	90.2 ± 8	<0.001
SBP (mmHg)^†^	116 ± 13	111 ± 11	121 ± 13	<0.001
DBP (mmHg)^†^	75 ± 9	71 ± 8	78 ± 9	<0.001
AST (IU/L)^†^	29 ± 17	26 ± 14	32 ± 19	<0.001
ALT (IU/L)^†^	31 ± 29	25 ± 25	38 ± 31	<0.001
GGT (U/L)^†^	55 ± 83	41 ± 70	71 ± 94	<0.001
Triglyceride (mg/dL)^†^	144 ± 111	100 ± 56	194 ± 135	<0.001
HDL (mg/dL)^†^	53 ± 13	57 ± 12	48 ± 11	<0.001
Glucose (mg/dL)^†^	99 ± 20	91 ± 8	108 ± 25	<0.001
FIB-4^†^	1.11 ± 0.6	1.07 ± 0.53	1.15 ± 0.68	<0.001
NFS^†^	-2.28 ± 1.2	-2.59 ± 1.09	-1.93 ± 1.21	<0.001
Liver stiffness (kPa)^†^	2.33 ± 0.58	2.27 ± 0.58	2.4 ± 0.57	<0.001
Sonographic fatty liver	2,963 (47.7)	1,024 (31.1)	1,939 (66.3)	<0.001
Significant hepatic fibrosis	411 (6.6)	129 (3.9)	282 (9.6)	<0.001
Advanced hepatic fibrosis	102 (1.6)	25 (0.8)	77 (2.6)	<0.001

Data are expressed as number (percent). ^†^Data are shown as mean ± standard deviation. Metabolically healthy group was defined as having less than two metabolic risks and not having diabetes. AST, aspartate transaminase; ALT, alanine transaminase; BMI, body mass index; DBP, diastolic blood pressure; FIB-4, fibrosis-4 index; GGT, γ-glutamyl transferase; HDL, high-density lipoprotein; MRE, magnetic resonance elastography; NFS, non-alcoholic fatty liver disease fibrosis score; SBP, systolic blood pressure.

### Comparison of diagnostic performance for advanced fibrosis between fibrosis-4 and NAFLD fibrosis score in the average-risk group

**FIGURE 2:**
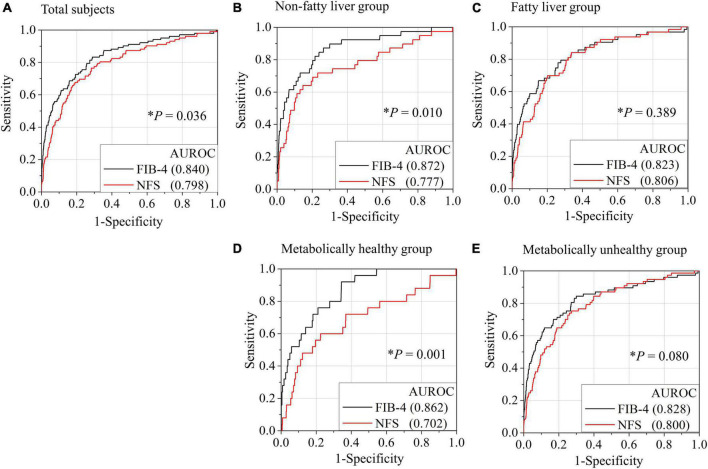
Comparison of AUROCs for advanced hepatic fibrosis (≥ 3.6 kPa) between FIB-4 and NFS based on the presence of fatty liver or metabolic status. Receiver operating characteristic (ROC) curves for the diagnosis of advanced fibrosis by FIB-4 or NFS in all subjects **(A)**, non-fatty liver group **(B)**, fatty liver group **(C)**, metabolically healthy group **(D)**, and metabolically unhealthy group **(E)**. A metabolically healthy state was defined as having less than two metabolic risks and not having diabetes. *P-value when the ROC curve by FIB-4 was compared with the ROC curve by NFS. NFS, NAFLD fibrosis score; FIB-4, fibrosis-4 index; AUROC, area under the receiver operating characteristic curve.

The AUROC of FIB-4 for the diagnosis of advanced hepatic fibrosis in the average-risk group was higher than that in the NFS (0.840 in FIB-4 vs. 0.798, *P* = 0.036) (Figure 2A). A comparison of AUROCs of FIB-4 and NFS in the fatty-liver group (Figure 2C) and the metabolically unhealthy group (Figure 2E) did not show any difference in the diagnostic performance of FIB-4 and NFS. However, the AUROCs of FIB-4 in the non-fatty liver group (0.872 in FIB-4 vs. 0.777 in NFS, *P* = 0.010) and the metabolically healthy group (0.862 in FIB-4 vs. 0.702 in NFS, *P* = 0.001) were significantly higher than those of NFS (Figures 2B,D). FIB-4 was found to be better for diagnosing advanced hepatic fibrosis than NFS in the average-risk group, because a considerable number of metabolically healthy (52.9%) subjects were included in this category.

### Sensitivity of fibrosis-4 and NAFLD fibrosis score to screen advanced fibrosis in the average-risk group

**FIGURE 3:**
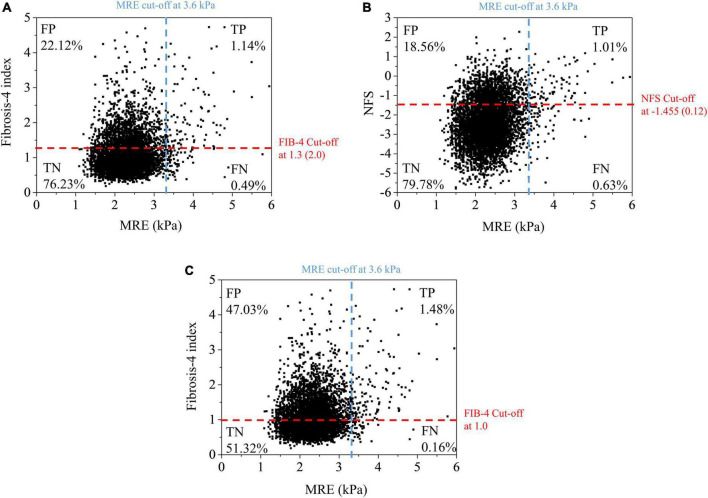
Scatter plot of liver stiffness measured by MRE vs. fibrosis-4 index **(A,C)** at different cut-off values and NFS **(B)**. Pearson correlation coefficient: MRE – FIB-4 (0.232), MRE – NFS (0.192). FN, false negative; FP, false positive; MRE, magnetic resonance elastography; NFS, NAFLD fibrosis score; FIB-4, fibrosis-4 index; TN, true negative; TP, true positive.

When we used the current cut-off of FIB-4 [1.3 (2.0)] and NFS [–1.455 (0.12)] for diagnosing advanced fibrosis (MRE value ≥ 3.6 kPa), the sensitivity and positive predictive value (PPV) were found to be low under most conditions (Figure 3 and Table 2). FIB-4 and NFS had low sensitivity (69.6 and 61.4%, respectively). In addition, the sensitivity of NFS further decreased in metabolically healthy (44.0%) and non-fatty liver (59.0%) groups when we applied the current low cut-off. The diagnostic performance of FIB-4 for advanced hepatic fibrosis was evaluated at different cut-off values (Table 3). As the cut-off value was increased from 0.9 to 1.3, the sensitivity decreased from 92.2 to 75.5% in the average-risk group. When the low cut-off of FIB-4, used as a first screening test for advanced hepatic fibrosis in the average-risk group, was lowered to 1.0, the sensitivity was 90.2% and the negative predictive value was 99.7%.

**TABLE 2:** Comparison of FIB-4 and NFS for diagnostic performance of advanced hepatic fibrosis in various groups.

	Advanced fibrosis* *n* (%)	Sensitivity (%)	Specificity (%)	PPV (%)	NPV (%)
** FIB-4 to use low cut-off; 1.3 (2.0)**					
Total subjects	102 (2.6)	69.6	77.5	4.9	99.3
Non-fatty liver group	39 (1.2)	71.8	76.7	3.6	99.6
Metabolically healthy group	25 (0.8)	64.0	78.2	2.2	99.6
Fatty liver group	63 (2.1)	68.3	78.4	6.4	99.1
Metabolically unhealthy group	77 (2.6)	71.4	76.7	7.7	99.0
** NFS to use low cut-off; –1.455 (0.12)**					
Total subjects	102 (2.6)	61.4	81.1	5.2	99.2
Non-fatty liver group	39 (1.2)	59.0	82.6	4.0	99.4
Metabolically healthy group	25 (0.8)	44.0	89.0	3.0	99.5
Fatty liver group	63 (2.1)	62.9	79.5	6.2	99.0
Metabolically unhealthy group	77 (2.6)	67.1	72.1	6.1	98.8

*Prevalence of subjects with advanced hepatic fibrosis among total subjects (*n* = 6,215), and the non-fatty liver (*n* = 3,252), metabolically healthy (*n* = 3,291), fatty liver (*n* = 2,963), and metabolically unhealthy (*n* = 2,924) groups. FIB-4, fibrosis-4 index; MRE, magnetic resonance elastography; NFS, non-alcoholic fatty liver disease fibrosis score; NPV, negative predictive value; PPV, positive predictive value.

**TABLE 3:** Comparison of diagnostic performance for advanced hepatic fibrosis (MRE cut-off values ≥ 3.6) according to the various cut-off values of FIB-4.

	Sensitivity	Specificity	PPV	NPV	Accuracy
**FIB-4 to use low cut-off; 1.3 (2.0)**					
Total subjects	69.6	77.5	4.9	99.3	77.4
Metabolically healthy group	64.0	78.2	2.2	99.6	78.1
**FIB-4 to use low cut-off; 0.9**					
Total subjects	92.2	42.1	2.6	99.7	42.9
Metabolically healthy group	100.0	43.6	1.3	100	44.0
**FIB-4 to use low cut-off; 1.0**					
Total subjects	90.2	52.2	3.1	99.7	52.8
Metabolically healthy group	96.0	53.8	1.6	99.9	54.1
**FIB-4 to use low cut-off; 1.1**					
Total subjects	87.3	60.9	3.6	99.7	61.3
Metabolically healthy group	92.0	61.9	1.8	99.9	62.2
**FIB-4 to use low cut-off; 1.2**					
Total subjects	83.3	69.0	4.3	99.6	69.2
Metabolically healthy group	80.0	69.8	2.0	99.8	69.9
**FIB-4 to use low cut-off; 1.3**					
Total subjects	75.5	75.4	4.9	99.5	75.4
Metabolically healthy group	76.0	76.7	2.4	99.8	76.7

FIB-4, fibrosis-4 index; MRE, magnetic resonance elastography; NFS, non-alcoholic fatty liver disease fibrosis score; NPV, negative predictive value; PPV, positive predictive value.

### Sensitivity analysis for diagnostic performance of fibrosis-4 in patients with specific conditions

**FIGURE 4:**
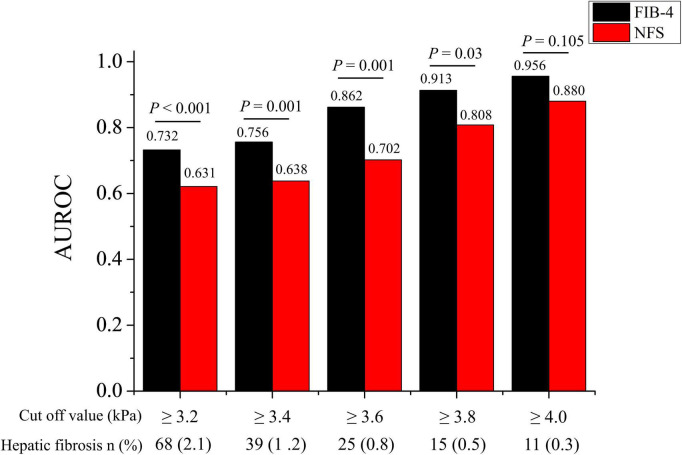
Comparison of AUROC for advanced hepatic fibrosis between FIB-4 and NFS in the metabolically healthy group at various MRE cut-off values (3.2–4.0 kPa). AUROC, area under the receiver operating characteristic curve; FIB-4, fibrosis-4 index; NFS, NAFLD fibrosis score.

Sensitivity analysis of the AUROCs of FIB-4 and NFS using various cut-off values for MRE (3.2–4.0 kPa) was performed (Table 4 and Figure 4). The AUROC of FIB-4 for hepatic fibrosis using various cut-off values for MRE (3.2–4.0 kPa) in an average-risk population was 0.709–0.896 (Table 4). Notably, the AUROC of FIB-4 for diagnosing hepatic fibrosis in the metabolically healthy group at different cut-off values for MRE was significantly higher than that of NFS, except for the cut-off value at 4.0 kPa (Figure 4). However, no difference in AUROCs was noted between FIB-4 and NFS in both the fatty liver and metabolically unhealthy groups at all cut-off values (Table 4).

**TABLE 4:** Comparison of AUROC for hepatic fibrosis between FIB-4 and NFS in various groups according to different MRE cut-off values.

	MRE cut-off	AUROC		*P*-value
		FIB-4	NFS	
Total	3.2	0.709	0.699	0.803
	3.4	0.751	0.745	0.761
	3.6	0.840	0.798	0.036
	3.8	0.867	0.843	0.174
	4.0	0.896	0.877	0.529
Non-fatty liver group	3.2	0.728	0.682	0.151
	3.4	0.759	0.706	0.129
	3.6	0.872	0.777	0.01
	3.8	0.925	0.867	0.122
	4.0	0.933	0.900	0.467
Metabolically healthy group	3.2	0.732	0.631	<0.001
	3.4	0.756	0.638	0.001
	3.6	0.862	0.702	0.001
	3.8	0.913	0.808	0.03
	4.0	0.956	0.880	0.105
Fatty liver group	3.2	0.704	0.704	0.706
	3.4	0.752	0.76	0.671
	3.6	0.823	0.806	0.389
	3.8	0.841	0.824	0.336
	4.0	0.876	0.860	0.622
Metabolically unhealthy group	3.2	0.695	0.688	0.984
	3.4	0.744	0.742	0.899
	3.6	0.828	0.800	0.08
	3.8	0.85	0.823	0.111
	4.0	0.878	0.852	0.346

Metabolically healthy group was defined as sharing less than two metabolic risks and not having diabetes. AUROC, area under the receiver operating characteristic curve; FIB-4, fibrosis-4 index; MRE, magnetic resonance elastography; NFS, non-alcoholic fatty liver disease fibrosis score.

## Discussion

Hepatic fibrosis, which is important risk factor in the prognosis of NAFLD patients is related not only to traditional TGF-β pathway, but also FOSL2, ADAM17, and angiotensin pathway (24–26). It is very important to identify high-risk groups by assessing the stage of hepatic fibrosis in suspected NAFLD patients (27). Currently, most guidelines recommend use of FIB-4 or NFS as a test for screening advanced liver fibrosis patients. We evaluated the diagnostic performance of FIB-4 and NFS in the average-risk group. The diagnostic performance of both FIB-4 (AUROC: 0.840) and NFS (AUROC: 0.798) was good even in the average-risk group. The diagnostic performance of FIB-4 was better than that of NFS for screening hepatic fibrosis in the average-risk group, while it was comparable to that of NFS in the metabolically healthy group. However, the diagnostic performance of the NFS decreased in the metabolically healthy and non-fatty liver groups. Because the current FIB-4 low cut-off has low accuracy and leads to false-negative results, it cannot be used as a single screening strategy in the average-risk group. Although diagnostic performance of FIB-4 was good, our large cohort reconfirmed considerable proportion of false-negative subjects with FIB-4 and very low PPV in the average-risk population. Currently, routine screening for advanced hepatic fibrosis is not recommended in general and average-risk populations. However, given that a considerable proportion of the general and average-risk population undergo health check-ups, a screening for hepatic fibrosis must be performed for those individuals who have already undergone medical check-ups based on a reasonable referral algorithm. Although the accuracy and positive-predictive value of FIB-4 are still not satisfactory, physicians can calculate FIB-4 using the pre-existing health check-up test item at no additional cost. FIB-4 can be a very attractive first screening method in a medical check-up setting, because additional sequential test can back-up the low positive-predictive value. Therefore, if we can increase the sensitivity of FIB-4 by adjusting the low cut-off values, FIB-4 can become the most cost-effective first-line screening test in average-risk populations.

FIB-4 and NFS have different variables because of different disease entities of the target population at the time of development. Clinical characteristics between two cohorts are very different only when their average BMIs (25 vs. 32) are compared (28, 29). Therefore, metabolic components such as BMI and the presence of impaired fasting glycemia or diabetes were included as variables only in the NFS formula. Consequently, the diagnostic performance of NFS is more easily affected by the metabolic status of the target population than that of FIB-4.

Interestingly, FIB-4 as an indicator of hepatic fibrosis worked well when applied to not only the fatty liver group (AUROC = 0.823) but also the non-fatty liver group (AUROC = 0.872) or metabolically healthy group (AUROC = 0.862). Conversely, the AUROCs of NFS in the non-fatty liver group (AUROC = 0.777) and metabolically healthy group (AUROC = 0.702) were lower or comparable to those in the fatty-liver group (AUROC = 0.800). Both FIB-4 and NFS have been validated well in patients with fatty liver or metabolic unhealthy. So, it makes sense that all of FIB-4 and NFS work well in subjects with fatty liver or metabolic unhealthy. However, the validation of FIB-4 and NFS especially in non-fatty liver or metabolic healthy has been very limited. Our results showed the AUROC of FIB-4 in metabolic healthy was even higher than that in metabolic unhealthy. The inverse tendency was also observed in NFS. Moreover, nearly 53% of total subjects were metabolically healthy. That is why the AUROC of FIB-4 was also significantly higher than that of NFS in all subjects (0.840 in FIB-4 vs. 0.798 in NFS, *P* = 0.036). Therefore, FIB-4 use should be recommended for screening subjects with hepatic fibrosis burden in average-risk groups.

Careful interpretation of the results obtained is required for several reasons. Most importantly, hepatic societies do not recommend routine screening for hepatic fibrosis in subjects without a risk of advanced hepatic fibrosis. We completely understand the concerns about unnecessary examinations. However, a non-negligible number of subjects with hepatic fibrosis (0.3–2.1%; MRE cut-off value = 3.2–4.0 kPa) were present in our cohort, although they were metabolically healthy and had no risk of viral and alcoholic hepatitis. The prevalence of hepatic fibrosis in all subjects was 0.9–3.9% (MRE cut-off value = 3.2–4.0 kPa). Moreover, the global hepatic fibrosis burden is gradually increasing owing to an increase in metabolic diseases. Therefore, preliminary studies for screening subjects with the hepatic fibrosis burden in the general and average-risk populations are needed. Our study is the first step in this regard.

Further study of the current cut-off values of FIB-4 for the average-risk group is needed. The current cut-off values proposed by McPherson et al. were originally optimized for screening subjects with advanced hepatic fibrosis among high-risk groups, such as those with fatty liver, viral hepatitis, and significant alcohol intake. A recent study reported that the FIB-4 and NFS have low accuracy for screening liver fibrosis in the general population (11). They pointed out the lower PPV for advanced fibrosis (LSM ≥ 12 kPa) by using a low cutoff value of FIB-4 (1.90% vs. 23.97%) and NFS (2.57% vs. 29.27%) in general population, compared to those in the high risk group. PPV and NPV are affected by the prevalence of disease. In the case of general population, whose disease prevalence is low, the low PPV is expected. In our study on average-risk subjects, similar PPV (4.9% in FIB-4, 5.9% in NFS) was observed when the current cut-off values were used. Non-negligible false-positive rates in FIB-4 (22.12%) and (18.56%) are observed (Figure 3). The accuracy of the current FIB-4 low cut-off was not satisfactory to be used as a single screening strategy in the average-risk group.

However, the screening strategy in the general population should focus on not missing patients with high hepatic fibrosis burden and finding subjects who can be managed through intensive lifestyle modification at the primary care center. If we consider this purpose, then we believe that the current low PPV and high false-positive rate can be tolerated, and even the lower cut-off values with relaxed standard are more appropriate for the general population. Shah et al. also suggested that a cut-off of 1.0 in FIB-4 can be appropriate for a primary care referral pathway (30). Our data also showed an increase in sensitivity (69.6–90.2% in total subjects; 64.0–96.0% in the metabolically healthy group) and comparable PPV and NPV when the cut-off value of 1.0 was used instead of using the previous optimal cut-off value [1.3 (2.0)], although more subjects without hepatic fibrosis were identified as positive (47.03% vs. 22.12%) (Figure 3). It is difficult to suggest what is the optimal cut-off value of FIB-4 for screening advanced hepatic fibrosis in general population based on our study alone. Another validation study for the new cut-off value and the socioeconomic assessment for application of FIB-4 for screening advanced hepatic fibrosis in general population should be needed in the future.

This study has some limitations as well. First, MRE, not liver biopsy, was performed to evaluate the degree of hepatic fibrosis. When comparing the diagnostic performance, the use of gold standard methods such as liver biopsy is necessary. However, it is impossible to routinely perform such methods in a health check-up setting. In this study, we evaluated MRE data because it is the most reliable non-invasive diagnostic method for estimating liver stiffness. Second, the proportion of men was higher than that of women in the study cohort. Moreover, there is a possibility that people concerned with liver health were more likely to be included in study, because MRE was offered as an additional option to be tested with their own expense. There is a possibility of selection bias. Large number of excluded subjects (*n* = 2,249) due to missing data can be another source of selection bias. Nevertheless, the overall prevalence of hypertension (26.4%), DM (9.2%), and metabolic syndrome (23.2%) was comparable to that in the general population. Third, there is no consensus on MRE cut-off values for advanced hepatic fibrosis. In this study, an MRE cut-off value of 3.6 kPa was used for advanced fibrosis. However, a similar result was obtained when the MRE cut-off value was set at either 3.2 or 4.0 kPa in the sensitivity analysis. Therefore, we believe that our results are reliable regardless of the cut-off values.

In conclusion, the AUROCs of FIB-4 and NFS for advanced fibrosis did not differ between the metabolically unhealthy and fatty liver groups. However, the AUROC of FIB-4 for advanced fibrosis was higher than that of NFS, particularly in the metabolically healthy and non-fatty liver groups. As more than half the average-risk population were consisted of metabolically healthy individuals, FIB-4 showed better performance for diagnosing advanced fibrosis when applied to the whole group with average-risk. It is recommended to use FIB-4 rather than NFS, when screening for hepatic fibrosis in general population.

## Data availability statement

The original contributions presented in this study are included in the article/supplementary material, further inquiries can be directed to the corresponding author/s.

## Ethics statement

The studies involving human participants were reviewed and approved by the Institutional Review board (IRB No. HY-2021-04-001-001) of Hanyang University Hospital. The ethics committee waived the requirement of written informed consent for participation.

## Author contributions

DJ: concept, design, and interpretation of data. EY, JL, MK, SC, and E-HN: data collection and management. HP: writing of the manuscript. DJ and E-HN: supervision. All authors read and approved the manuscript.
